# sRNAbench and sRNAtoolbox 2019: intuitive fast small RNA profiling and differential expression

**DOI:** 10.1093/nar/gkz415

**Published:** 2019-05-22

**Authors:** Ernesto Aparicio-Puerta, Ricardo Lebrón, Antonio Rueda, Cristina Gómez-Martín, Stavros Giannoukakos, David Jaspez, José María Medina, Andreja Zubkovic, Igor Jurak, Bastian Fromm, Juan Antonio Marchal, José Oliver, Michael Hackenberg

**Affiliations:** 1 Dpto. de Genética, Facultad de Ciencias, Universidad de Granada, Campus de Fuentenueva s/n, 18071 Granada, Spain; 2 Lab. de Bioinformática, Centro de Investigación Biomédica (CIBM), PTS, Avda. del Conocimiento s/n, 18100 Granada. Spain; 3 Genomics England, Charterhouse Square, London EC1M 6BQ, UK; 4 Department of Biotechnology, University of Rijeka, Croatia; 5 Science for Life Laboratory, Department of Molecular Biosciences, The Wenner-Gren Institute, Stockholm University, Stockholm, Sweden; 6 Department of Human Anatomy and Embryology, Institute of Biopathology and Regenerative Medicine, Excellence Research Unit ‘Modeling Nature' (MNat), University of Granada, Granada, Spain; 7 Instituto de Investigación Biosanitaria ibs.GRANADA, University Hospitals of Granada-University of Granada, Spain; Conocimiento s/n, 18100 Granada. Spain

## Abstract

Since the original publication of sRNAtoolbox in 2015, small RNA research experienced notable advances in different directions. New protocols for small RNA sequencing have become available to address important issues such as adapter ligation bias, PCR amplification artefacts or to include internal controls such as spike-in sequences. New microRNA reference databases were developed with different foci, either prioritizing accuracy (low number of false positives) or completeness (low number of false negatives). Additionally, other small RNA molecules as well as microRNA sequence and length variants (isomiRs) have continued to gain importance. Finally, the number of microRNA sequencing studies deposited in GEO nearly triplicated from 2014 (280) to 2018 (764). These developments imply that fast and easy-to-use tools for expression profiling and subsequent downstream analysis of miRNA-seq data are essential to many researchers. Key features in this sRNAtoolbox release include addition of all major RNA library preparation protocols to sRNAbench and improvements in sRNAde, a tool that summarizes several aspects of small RNA sequencing studies including the detection of consensus differential expression. A special emphasis was put on the user-friendliness of the tools, for instance sRNAbench now supports parallel launching of several jobs to improve reproducibility and user time efficiency.

## INTRODUCTION

Small RNA profiling by means of miRNA-seq (or small RNA-seq) is a key step in many study designs because it often precedes further downstream analysis such as screening, prediction, identification and validation of miRNA targets or biomarker detection (1,2). Many different tools are available for the analysis of small RNA high-throughput sequencing data such as miRDeep2 (3), miRge 2.0 (4), ShortStack (5), SeqBuster (6), sRNAbench (7) and miRTrace (8) which implements a new approach to quality control. Generally, the tools focus on certain aspects of small RNAs and are not integrated into independent pipelines for downstream analysis. In 2015, we introduced sRNAtoolbox (9), a collection of small RNA research tools built around sRNAbench, providing different downstream analysis including consensus differential expression, target prediction and analysis of unmapped reads by means of blast searches against general nucleotide databases.

The last few years have witnessed a further drop in sequencing cost that together with the advent of highly specialized service providers makes the generation of this kind of data accessible to a larger number of research groups. The increase in sequencing volume has been accompanied by the publication of new library preparation protocols, each of which involves specific pre-processing steps in the bioinformatics analysis. However, not all research groups can count on specialized staff or bioinformatics equipment, which is why flexible and user-friendly tools for small RNA research became even more valuable over the last years.

Here, we present the latest version of sRNAtoolbox, featuring key additions to sRNAbench and sRNAde. Apart from customizable preprocessing, sRNAbench now implements automatic processing of the five most used library preparation protocols including UMI-based (Unique Molecular Identifier) protocols and the detection of putative sequence variants. The scope was notably increased by including new reference genomes from Ensembl (release 91), bacteria and virus collections from NCBI and microRNA reference sequences from MirGeneDB. Additionally, in order to improve reproducibility and ease of use, a *batch mode* was developed to allow profiling of several samples at once using the same set of parameters. As for sRNAde, now consensus results for five differential expression methods are calculated together with improved visualizations of several quality and mapping statistics.

## WHAT’S NEW?

Since sRNAtoolbox web-server has previously been described (9), we briefly present main novelties and changes in this section. More detailed descriptions can be found in the *Data and methods* section.

- **sRNAbench batch mode:** users can now provide an unlimited number of reads files through upload, URLs or SRA Run accessions. In this way, parameters only need to be specified once and are applied to all input data.
- **Reanalysis of provided files:** All provided files can be reanalysed without reuploading to the server.
- **New sRNAbench features:** Optional quality control of fastq input, detection of sequence variants, direct availability of 6 different library preparation protocols, UMI (Unique Molecular Identifier) protocols are supported, isomiR classification can be made hierarchical (each read belongs to only one category) or fuzzy (each read can belong to several categories), input format is automatically detected to prevent inconsistent file extensions and improved feedback so most frequent input errors can be corrected by the user.
- **Visualization of genome mapped reads:** The jBrowse instance to visualize the genome mappings was replaced by links to UCSC Genome Browser or Ensembl track hubs. Additionally, direct downloads to bedGraph, bigWig and bed files are provided so they can be analysed using specialized software like the Integrative Genome Viewer (10).
- **Differential expression:** We added two additional methods to detect differentially expressed microRNAs: a Student's t-test and DESeq2 (11) for a total of 5 different methods. Each method has its own output page which includes interactive heatmaps (12), box-plots and volcano plots to visualize differences in expression values between two groups. The consensus differentially expressed microRNAs are visualized by means of UpsetR (13), an alternative to Venn diagrams. By default, adjusted read counts (to address multiple mapping) are used to generate the expression matrixes, but matrixes for other multiple mapping methods can be found in the downloadable results.
- **Consensus target detection:** The original miRconstarget was split into two, one tool for animals and one for plants. A simple seed detection method several folds faster than the other three (miranda, PITA and TargetSpy) was added to the animal tool.
- **Scope:** Genome sequences and annotations are automatically derived from Ensembl (14). Current version of sRNAtoolbox contains 90 genome assemblies and several virus and bacteria collections obtained from NCBI (15).
- **Reference sequences:** microRNAs for all species included in miRBase (16) or MirGeneDB (17) can be profiled regardless of genome availability.
- **liqDB**: sRNAbench is now connected to liqDB, a small RNA database for liquid biopsy studies (18), i.e. sRNAbench output can be used to compare against liqDB profiles.

## DATA AND METHODS

### Input data

Input files can be uploaded to our server, be provided as URLs or as SRA Run IDs (19). For URLs or SRA run identifiers, several files can be merged together by joining them using colons (:). For example SRR2105509:SRR2105510 would merge both SRA runs into a single job. In the previous sRNAbench version, the input format was detected based on the file extension only, i.e. *.fastq for fastq format, *.fa for fasta format and *.rc for read count format. Because sRNAbench jobs could fail due to an incorrect extension, we included now an automatic detection of the input format to prevent those errors. Automatic detection of most common separators in read-count encoded fasta files has also been implemented.

### Quality control

Two quality filters have been implemented in sRNAbench for fastq files. The ‘mean’ method calculates the average PhredScore of the adapter-trimmed read, filtering out those below a certain threshold. The ‘min’ method is stricter as it sorts out any read with at least one position below the provided threshold.

### MicroRNA profiling, genome and library mode

Expression values can be obtained either using genome or library mode. In genome mode, reads are first mapped to the corresponding assembly and genome annotations of the reference sequences are used to obtain the expression values. In library mode, reads are mapped directly against the reference sequences. Both methods are described in detail in the original sRNAbench paper (7). MicroRNA expression profiles can be obtained for all species contained in miRBase or MirGeneDB by means of the library mode. It is important to note that expression files generated with sRNAbench will list all copies of a microRNA, and therefore the name of a mature microRNA can appear several times. However in an additional column we specify the genome position or precursor name, which makes each line unique. Two different methods are provided for multiple mapping, (i) adjusting the read count by the number of times the read maps to the genome or reference sequences and (ii) assign each read only once to the reference sequence with the highest expression (single assignment) (see (7) for more details). The prediction of novel microRNAs was described before in the sRNAbench paper (7) and a more detailed description is available in the manual as well.

### Genome mapping, bedGraph, bigWig and bed files

Adapter trimmed and quality filtered reads are mapped to the genome by means of bowtie1 (20). By default, bowtie seed alignment is used in order to detect isomiRs (with seed length of 20 nt) and reads are only used if they have at most 10 mappings to the genome. The best mappings are retained as explained before (21). Both parameters can be changed by the user. For the prediction of novel microRNAs, we recommend ‘full read alignment’ and not allowing mismatches. Some putatively interesting small RNAs like yRNAs have many copies in the genome, and therefore the maximum number of allowed mappings might need to be increased in such cases.

Reads with more mappings to the genome than specified by this threshold are not used for expression profiling but will appear as a separate category in the genome mapping plots. Those reads are labelled *Highly Redundant* reads and are marked with the postfix (_*HR*).

Downloadable bedGraph files are generated summing the reads that map to a certain position. Note that in this way, each read counts fully at each position it maps (full read assignment). In the standalone version, the user can chose to adjust for multiple mappings. BedGraph files are generated irrespectively of the strand and for both strands separately (three files in total). Sometimes, it might be interesting to analyse the genome distribution as a function of the read length (20).Therefore, we provide the bedGraph files for different length intervals: 19 nt–23 nt and all lengths for animals and 19 nt–23 nt, 24 nt and all lengths for plants given that 24 nt long reads have a very well described function in plants (22). The bedGraph files are then converted to bigWig files using the UCSC tool *bedGraphToBigWig* (23). Finally, the bedGraph files are screened and continuously mapped regions are merged together into a six-column bed file. The provided score indicates the highest expression value of the region as not all positions in a continuously mapped region will have the same expression values.

### Single nucleotide variants

Single nucleotide variants (SNV) are detected based on reported mismatches. They can be due to Single Nucleotide Polymorphisms (SNPs), somatic mutations, RNA editing, sequencing or Taq polymerase errors. Therefore, when those sequence variants are analysed, strict quality control parameters should be used to control for the effect of sequencing errors and other technical artefacts. As the quality scores (Phred Scores) are not used for the detection of SNVs, this analysis can be performed for all accepted input formats. The sequence variants are detected at the level of precursor sequences, giving for each variant the precursor name, the variant type, the position, the number of mapped reads and the number of reads containing the variant.

### isomiRs

The original sRNAbench version implemented only a hierarchical isomiR classification, i.e. each read is classified as only one isomiR type: canonical sequence, canonical sequence with nucleotide changes, non-templated additions, 5’ and 3’ length variants or multiple length variants (in this hierarchical order). However, a read can have both, sequence and length variation. Therefore, we now added the possibility to explore the impact of a fuzzy classification. sRNAbench output files can be used to convert the isomiR data into standardized formats as proposed by the miRTop community (https://www.biorxiv.org/content/10.1101/505222v1, https://github.com/miRTop/mirtop).

### Differential expression

The differential expression program sRNAde has undergone profound changes to provide both, an extensive summary of the whole study and the detection of consensus differential expression applying edgeR (24), DESeq (25), DESeq2 (11), NOISeq (26) and Student's *t*-test. Additionally, each method now has an individual page to explore the different results as well as the consensus. The output page was separated into 5 sections:

- **Results Summary:** The number of differentially over and underexpressed microRNAs per method and visualizations for the distribution of detected RNA types like miRNAs, tRNAs, rRNAs etc.
- **Preprocessing/QC:** Summary of preprocessing (adapter trimmed reads, filtered reads) and read length distribution which allows to detect the presence of certain types of small RNAs (peak around 21nt corresponding to miRNAs) or artefacts like the presence of adapter dimers (reads with length 0).
- **Mapping statistics:** overview of the number of mapped and assigned reads.
- **miRNA and isomiR statistics:** boxplots with number of detected miRNAs, link to microRNA sequence variant analysis and isomiR statistics.
- **Differential expression:** links to the individual output pages of the five DE methods, consensus tables and its graphical representation by means of UpSet plots (equivalent to Venn diagrams).

Furthermore, sRNAde provides now three different methods to address the multiple mapping problem: (i) full read count assignment (the full read count is assigned to all reference sequences or genome positions), (ii) adjusted read counts (divide the read count by the number of mappings) and (iii) single assignment, i.e. assign the read only once to the most expressed reference sequence.

### Working examples

**Figure 1.**
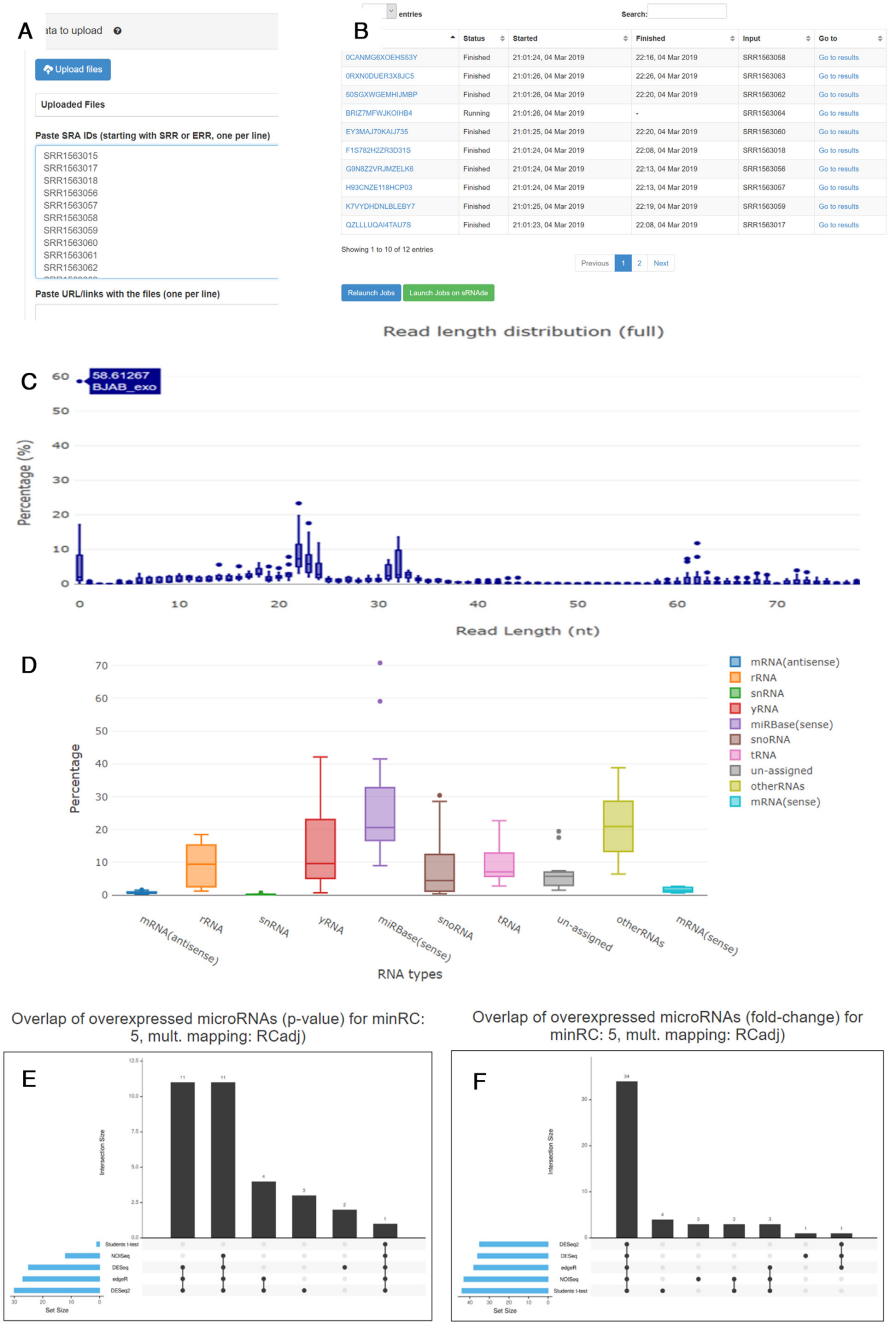
(**A** and **B**) The interface of the sRNAbench batch mode module and the primary result table, (**C**) The read length distribution as box-plot, i.e. the distribution of read fraction as a function of read length, (**D**) the distribution of different RNA types in the study, (**E**) the intersection of up-regulated microRNAs between the different methods and (**F**) the intersection of microRNAs with higher fold-changes than 2.

To demonstrate the usefulness and functionality of the newly implemented features we will concentrate on the sRNAbench (batch mode) and sRNAde tools. The batch mode is a novel extension of sRNAbench which first requests the upload of the sequencing data. We strongly recommend depositing sequencing data on an accessible server and providing the URLs by means of the corresponding textbox. The sequencing data can also be uploaded through the browser or specified by means of SRA run IDs. To illustrate the analysis of data from the public SRA (Sequence Read Archive) repository, we used the SRP046046 (27) study, which can be accessed through this page: https://www.ncbi.nlm.nih.gov/Traces/study/?acc=SRP046046. This study has 12 different biological samples and one run per sample. After downloading the samples annotations (*RunInfo Table*), they can be imported into any spreadsheet program. In this way, the column with the run names (starting with *SRR*) can be easily copied and pasted into the sRNAbench (batch mode) interface (see Figure 1A). After this step, the user needs to provide information regarding the species (human) and library preparation protocol (Illumina). For each sequencing data file, a separate sRNAbench job will be created. The current state of the jobs will be shown to the user on the sRNAbench (batch mode) output page (see Figure 1B). Once all jobs have finished, the results of the individual sRNAbench jobs can be used as input for sRNAde (study summarizing and differential expression tool). In order to use sRNAde, a group label needs to be assigned to each sample to indicate the condition (such as healthy, cancer, treated, etc). The output page includes a button that will take the user through this process. Note that input samples and group information can be provided in other ways through the sRNAde page. The general structure of the sRNAde output page was previously described in the ‘Data and methods’ section, so here we will highlight some of the newly implemented features that will help users to better interpret their data. The read length distribution of adapter-trimmed reads (in ‘Preprocessing/QC’ section of sRNAde output page) contains valuable information to spot possible artefacts in the library preparation. By moving the mouse cursor over the boxplots, the values of the extreme points are depicted. Figure 1C shows that in general the number of adapter-dimer reads (the adapters have ligated directly without a fragment in between) are below 20%, however one sample (BJAB exosomes, SRR1563017) shows nearly 60% of adapter-dimers, which can indicate some issues in the library preparation like low RNA input. In general, clear peaks corresponding to the lengths of certain RNA types should be distinguishable: microRNAs should form a narrow peak around 21–22nt and tRNAs are known to generate fragments around 18 nt and between 32 and 33 nt. If no peaks are distinguishable or if they are very smeared out, this can indicate low RNA quality (high degradation). In Figure 1C we can observe the existence of a broad peak around the length of microRNA precursor sequences or full length tRNAs. Figure 1D shows the distribution of RNA types in the study. This graphic enables the user to obtain information about the relative quantities of miRNAs or other RNA molecules like yRNA tRNA, snoRNA or rRNA. Furthermore, the dispersion of relative frequencies of a given RNA type over the different samples can be observed. For example, the percentage of microRNAs varies between 10% and 70% in this case.

Figure 1E shows the overlap of differentially expressed microRNA between the five methods and Figure 1F depicts the overlap of microRNAs with a log2 fold-change higher than 1 or lower than -1. Note that to avoid division by 0, we add the value of 1 to the expression values. This also leads to the fact that microRNAs with extremely low expression values are less likely to produce high fold-changes due to chance alone. It can be seen that the overlap using the fold-change is very high (34 out of 49). Notice that the miRNA fold-change only depends on the normalized values of the read count input matrix (same for all methods). Therefore, the high overlap seems to imply that the normalization methods have a rather moderate impact on the fold-changes. On the other hand, there is only 1 out of 32 microRNA which shows statistically over-expression in all five methods mainly because Student's *t*-test and NOISeq seem to be much stricter. DEseq, DESeq2 and edgeR are the methods with the highest overlap (11 out of 32). This shows that the way the *P*-values are calculated strongly impacts the detection of differentially expressed microRNAs.

## CONCLUSIONS AND OUTLOOK

Over the last years the user feedback was crucial for the evolution of sRNAtoolbox. Several of the new features and species were included upon user request. We encourage users to send feedback of any type to continue improving this collection of small RNA research tools. Upcoming improvements include, among other features, new annotations, support for user-customizable synthetic spike-ins and improved prediction of novel microRNAs.

## DATA AVAILABILITY

https://arn.ugr.es/srnatoolbox/
